# *Capnocytophaga canimorsus* Mycotic Aortic Aneurysm After a Dog Bite

**DOI:** 10.1016/j.ejvsvf.2022.04.005

**Published:** 2022-04-29

**Authors:** Robert H.A. Berndsen, Pim B.J.E. Hulshof, Maurits P.A. van Meer, Ben R. Saleem, Vincent P.W. Scholtes, René M. The, Vincent Jongkind, Kak Khee Yeung

**Affiliations:** a Department of Surgery, Amsterdam Cardiovascular Sciences, Amsterdam University Medical Centres, Amsterdam, the Netherlands; b Department of Internal Medicine, Amsterdam University Medical Centres, Vrije Universiteit, Amsterdam, the Netherlands; c Department of Medical Microbiology, Amsterdam University Medical Centres, Amsterdam, the Netherlands; d Department of Surgery, Division of Vascular Surgery, UMCG, Groningen, the Netherlands; e Department of Surgery, St Jansdal Ziekenhuis, Harderwijk, the Netherlands

**Keywords:** Biological graft, IS-pro, Mycotic aortic aneurysm, Open repair

## Abstract

**Introduction:**

Mycotic aortic aneurysm is defined as dilatation of the aortic wall due to infection caused by a variety of microorganisms and is associated with high mortality rates. This case report describes a patient with a rapid growing mycotic infrarenal aneurysm caused by *Capnocytophaga canimorsus* following a dog bite.

**Report:**

A 61 year old male professional dog handler presented with a history of progressive abdominal pain and constitutional symptoms. He had been bitten by a Pit Bull Terrier dog that was attacking a young girl three weeks prior to the onset of complaints. Investigations revealed a mycotic infrarenal aortic aneurysm that grew 0.5 cm in only three days. Open surgical repair consisting of an infrarenal aorto-aortic bypass with a 21 mm × 15 cm bovine bioprosthesis was performed successfully. All cultures and biopsies were negative and the subsequent 16S–23S rRNA intergenic spacer region based polymerase chain reaction (IS-pro) technique revealed *C. canimorsus*, a Gram negative bacterial pathogen that lives as a commensal in the gingival flora of dogs and cats that can cause a variety of severe infections, as the causative agent. Identification made it possible to treat the patient with eight weeks of intravenous followed by four weeks of oral antibiotics. At the last follow up over a year after surgery, the patient was symptom free, without infection and on ultrasound examination there were no signs of complications or aneurysm formation.

**Discussion:**

This case highlights *C. canimorsus* as a rare cause of a rapid growing mycotic aortic aneurysm following a dog bite. 16S–23S rRNA profiling (IS-pro) led to the identification of the bacterial pathogen. The use of biological grafts should be considered in the management of mycotic aortic aneurysms.

## Introduction

Mycotic aortic aneurysm is defined as dilatation of the aortic wall due to infection caused by microorganisms and represents 0.7%–3% of all aortic aneurysms.^1^ Most frequently, mycotic aneurysms are caused by Gram positive cocci such as *Staphylococcus aureus* and *Streptococcus pneumoniae* and Gram negative *Salmonella species.*^2^ However, a variety of rare pathogens have also been described to cause mycotic aneurysms.^2^ Identification of the causative pathogen is essential as adequate antibiotic therapy is crucial in their management. Molecular detection methods are increasingly being used to identify bacterial species that are difficult to culture *in vitro*. The 16S–23S rRNA intergenic spacer region based polymerase chain reaction (PCR) technique (IS-pro, inBiome, Amsterdam, The Netherlands) has certain advantages over the more commonly used 16S rRNA PCR with subsequent Sanger sequencing as it is fast and allows the identification of multiple species within one sample.^3^

This report describes a patient with a rapidly growing mycotic infrarenal aneurysm following a dog bite who underwent successful open repair with a bovine bioprosthesis; the subsequent 16S–23S rRNA intergenic spacer region based PCR (IS-pro) technique identified *Capnocytophaga canimorsus* as the bacterial pathogen.

## Case Report

A 61 year old male professional dog handler presented to a local emergency department with four days of progressive abdominal pain radiating to the lower back, fatigue, nausea, muscle strains, and a cold chill. His past medical history included hypertension and chronic obstructive pulmonary disease and he had a history of smoking (30 pack years). He had no history of any form of arterial disease; however, there were no prior imaging studies available that could indicate a pre-existing aortic aneurysm. The patient showed signs of colic like abdominal pains. Vital signs included pulse of 116/min, blood pressure of 129/80 mmHg, respiratory rate of 14, and temperature of 37.7°C. Physical examination revealed abdominal pain in the right lower quadrant. The abdominal aorta was not palpable and thus not painful. Laboratory evaluation revealed elevated inflammatory parameters with a white blood cell count of 23 x10^9^/L and C reactive protein (CRP) level of 264 mg/L. The haemoglobin level and pancreatic enzymes were normal.

**Figure 1:**
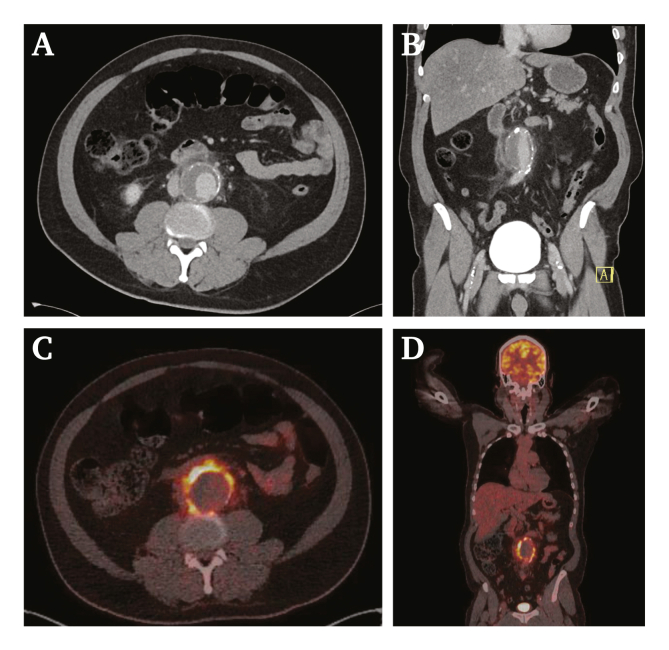
Computed tomography (CT) in axial (A) and coronal (B) view on admission demonstrating a 4.5 cm aortic aneurysm with inflammation of fatty tissue and fluid surrounding the aorta. ^18^F-fludeoxyglucose (FDG) positron emission tomography/low dose (ld) CT scan in axial (C) and coronal (D) view demonstrating significant heterogeneous FDG uptake of the aortic wall and an increase in diameter of the aortic aneurysm from 4.5 to 5.0 cm. FDG uptake was observed in retroperitoneal lymph nodes most likely reactive to the infected arterial wall. The spleen and bone marrow also showed weak uptake suggestive of an active infection. Physiological FDG uptake was seen in the brain and myocardium with signs of renal tracer excretion.

A computed tomography (CT) scan with intravenous contrast demonstrated a 4.5 cm infrarenal abdominal aortic aneurysm with signs of inflammation and fluid around the aneurysm down to the iliac vessels, suggestive of a mycotic aneurysm (Fig. 1A and B). The patient was admitted, and after blood culture sampling broad spectrum antibiotic therapy with amoxicillin/clavulanic acid was initiated. An ^18^F-fludeoxyglucose (FDG) positron emission tomography (PET)/low dose (ld) CT scan was performed three days after admission showing significant heterogeneous FDG uptake in the soft tissue surrounding the aneurysm and in surrounding lymph nodes without signs of increased uptake elsewhere (Fig. 1C and D). The aortic aneurysm diameter was now 5.0 cm, indicating a growth of 0.5 cm in only three days. After this finding, the patient was transferred to a university hospital with relevant expertise for further treatment. At this time the patient revealed he had superficial dog bite wounds on his hands after protecting a young girl from a dog attack three weeks prior to the onset of complaints. Owing to this finding, a canine bacterial pathogen was considered the most likely cause. Based on the clinical presentation, antibiotic therapy was widened to ceftriaxone and vancomycin.

**Figure 2:**
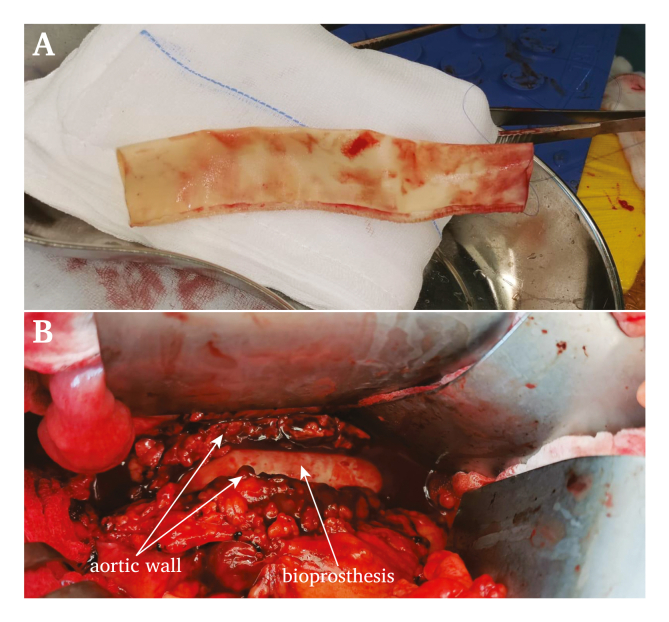
Intra-operative image of the bioprosthesis before (A) and after placement with the surrounding infected aortic wall (B).

Surgical repair was indicated and use of an autologous vein graft was considered; however, given the medical history of the patient and risk of increased morbidity this was not selected. Instead, open repair with a bioprosthesis was performed due to evidence suggesting a lower risk of graft re-infection than using a prosthetic graft.^4^^,^^5^ During surgery, the aortic wall was smooth, significantly thickened, and showed signs of inflammation and hypervascularisation. However, no debris or pus was found. A 21 mm × 15 cm bovine bioprosthesis (BioIntegral Surgical, Canada) was used for interposition and sutured to healthy infrarenal aorta (Fig. 2). Full aneurysm resection and debridement of all infected tissue was not performed because of the absence of debris or pus and because it would have significantly increased the operating time and blood loss. Multiple biopsies from the aortic wall and surrounding inflamed tissue were taken for Gram stain, culture, and histopathology after infrarenal clamping. The post-operative course was, besides a short period of gastroparesis, uncomplicated and the patient was discharged 10 days after surgery.

The Gram stain revealed leucocytes but no microorganisms. All cultures of blood and biopsies remained negative, possibly related to pre-operatively continued antibiotic treatment. Serology for *Treponema pallidum* and *Coxiella burnetii* were also negative. Subsequent 16S–23S rRNA intergenic spacer region based PCR (IS-pro)^3^ was performed that identified *C. canimorsus* in all biopsies and was considered the causative agent. Owing to negative cultures, antibiotic susceptibility testing could not be performed and empirical antibiotic treatment with ceftriaxone was continued.

Eight weeks after surgery, an ^18^F-FDG PET/ldCT showed a significant decrease in FDG uptake of the aortic wall. Intravenous ceftriaxone treatment was discontinued and administration of oral amoxicillin/clavulanic acid was initiated for four more weeks. After antibiotic therapy was finished the patient was in good condition and laboratory results (i.e., inflammatory markers, CRP, erythrocyte sedimentation rate, and leucocytes) did not show any signs of persistent infection. Follow up at six months and a year after surgery showed no complications and a good graft patency without dilatations. Further annual follow ups with abdominal ultrasound investigation were planned.

## Discussion

*Capnocytophaga canimorsus* is a fastidious and slow growing Gram negative fusiform bacterium with medium to long cells with tapered ends which lives as a commensal in the gingival flora of dogs (85%–100% as determined by PCR) and cats (15% as determined by culture). Transmission occurs by biting, scratching, or licking and can cause zoonotic disease in humans. *Capnocytophaga canimorsus* can resist phagocytosis by macrophages and killing by complement and polymorphonuclear leucocytes. Infections with *C. canimorsus* are rare (a reported incidence of 0.67 infections per million population in The Netherlands between 2003 and 2005^6^) and are associated with high mortality rates of 28%–30% due to bacteraemia and subsequent sepsis. To date, there have been four cases of mycotic aorto-iliac aneurysms described as caused by *C. canimorsus*, and in three of these cases the initial presentation was with a ruptured or contained ruptured aneurysm indicating a fulminant clinical course, in line with the case presented here.

In the management of mycotic aortic aneurysms, identification of the causative pathogen is essential to guide antibiotic therapy. However, in general, blood cultures of patients with mycotic aneurysms are negative in 15%–50% of cases and since peri-operative biopsies of infected tissue are usually taken under broad spectrum antibiotics, tissue cultures can also remain negative. In this case, the causative pathogen was discovered by the IS-pro molecular technique, which is based on the length and sequence polymorphisms of the 16S–23S ribosomal interspace regions of bacteria after using phylum specific fluorescently labelled PCR primers. Subsequently, the species specific generated “IS-pro peaks” are analysed by automated correlation with a validated database containing IS-pro peak information of more than 500 bacterial species.^3^ Alternatively, broad range 16S rRNA PCR with subsequent Sanger sequencing can be used to detect bacterial DNA and identify the bacterial species after performing a 16S rRNA sequence homology analysis in the NCBI GenBank. The advantage of IS-pro over 16S rRNA PCR and subsequent Sanger sequencing is that IS-pro is fast (results obtained within five hours) and can easily detect several species in the same sample.^7^ However, IS-pro identification remains limited to only its own validated database of bacterial pathogens.

In the presented case, a bovine bioprosthesis was used for surgical repair. If surgical repair is performed with prosthetic grafts such as polytetrafluoroethylene or Dacron (polyester) they can be considered infected or at least pose a risk of the development of a prosthetic vascular graft infection later on, which is associated with high mortality rates.^8^ Evidence suggests that the use of biological grafts (i.e., autologous grafts, allografts, and bioprostheses) can prevent graft infections and subsequent complications.^5^^,^^8^ This implies a potential advantage for limiting re-interventions and the duration of post-operative antibiotic treatment.

Currently there is a lack of consensus guidelines that guide the management of mycotic aortic aneurysms and evidence mostly originates from single centre retrospective studies. To enable comparative studies to provide a reliable analysis of treatment strategies and outcomes first there is a need for consensus on definitions and diagnostic criteria which was acknowledged recently by Sörelius et al.^9^ In the absence of consensus guidelines, and since there are significant variations in patient characteristics, diagnostic approaches and surgical and antibiotic treatment options, as this case highlights, a multidisciplinary and patient tailored approach including vascular surgeons, clinical microbiologists, and infectious disease experts is key to ensure the best management of patients with mycotic aortic aneurysms.^10^

In conclusion, the case of a rapid growing mycotic aortic aneurysm caused by the rare bacterial pathogen *C. canimorsus* after a dog bite that was successfully treated with open surgical repair including a bovine bioprosthesis is described. The 16S–23S rRNA intergenic spacer region based PCR (IS-pro) technique identified the causative pathogen, thus confirming the diagnosis and guiding post-operative antibiotic therapy.

## Conflict of interest

None.

## Funding

None
